# 1,4-Bis{3-[4-(dimethyl­amino)benzyl­ideneamino]prop­yl}piperazine

**DOI:** 10.1107/S1600536809045619

**Published:** 2009-11-04

**Authors:** Rui-Bo Xu, Xing-You Xu, Da-Qi Wang, Xu-Jie Yang, Shuan Li

**Affiliations:** a Department of Chemical Engineering, Huaihai Institute of Technology, Lianyungang 222005, People’s Republic of China; b Huaiyin Institute of Technology, Huaian 223003, People’s Republic of China; c College of Chemistry and Chemical Engineering, Liaocheng University, Shandong 252059, People’s Republic of China; d Materials Chemistry Laboratory, Nanjing University of Science & Technology, Nanjing 210094, People’s Republic of China

## Abstract

The mol­ecule of the title compound, C_28_H_42_N_6_, has site symmetry 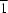 with the centroid of the piperazine ring located on an inversion center. The piperazine ring adopts a chair conformation. The benzene ring and propyl­piperazine are on opposite sides of the C=N bond, showing an *E* configuration.

## Related literature

For applications of Schiff base compounds, see: Basak *et al.* (2008 ▶); Jiang *et al.* (2008 ▶); Xu *et al.* (2008 ▶). For *N*,*N*′-disubstituted piperazine derivatives, see: Yogavel *et al.* (2003 ▶). For related structures, see: Paital *et al.* (2009 ▶); Thirumurugan *et al.* (1998 ▶).
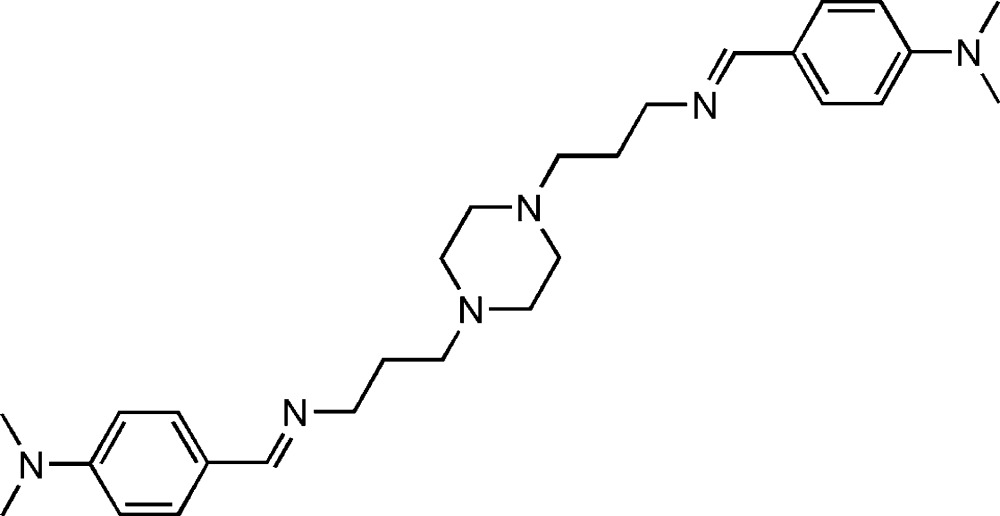


## Experimental

### 

#### Crystal data

C_28_H_42_N_6_
*M* *_r_* = 462.68
Monoclinic, 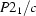

*a* = 17.599 (2) Å
*b* = 6.4146 (12) Å
*c* = 12.6643 (18) Å
β = 105.921 (3)°
*V* = 1374.8 (4) Å^3^
*Z* = 2
Mo *K*α radiation
μ = 0.07 mm^−1^
*T* = 298 K
0.15 × 0.09 × 0.07 mm

#### Data collection

Bruker SMART CCD area-detector diffractometer
Absorption correction: none
6788 measured reflections
2416 independent reflections
961 reflections with *I* > 2σ(*I*)
*R*_int_ = 0.088

#### Refinement

*R*[*F* ^2^ > 2σ(*F* ^2^)] = 0.095
*wR*(*F* ^2^) = 0.298
*S* = 1.34
2416 reflections
155 parameters
H-atom parameters constrained
Δρ_max_ = 0.24 e Å^−3^
Δρ_min_ = −0.17 e Å^−3^

### 

Data collection: *SMART* (Siemens, 1996 ▶); cell refinement: *SAINT* (Siemens, 1996 ▶); data reduction: *SAINT*; program(s) used to solve structure: *SHELXTL* (Sheldrick, 2008 ▶); program(s) used to refine structure: *SHELXTL*; molecular graphics: *SHELXTL*; software used to prepare material for publication: *SHELXTL*.

## Supplementary Material

Crystal structure: contains datablocks I, global. DOI: 10.1107/S1600536809045619/xu2643sup1.cif

Structure factors: contains datablocks I. DOI: 10.1107/S1600536809045619/xu2643Isup2.hkl

Additional supplementary materials:  crystallographic information; 3D view; checkCIF report

## Figures and Tables

**Fig. 1.**
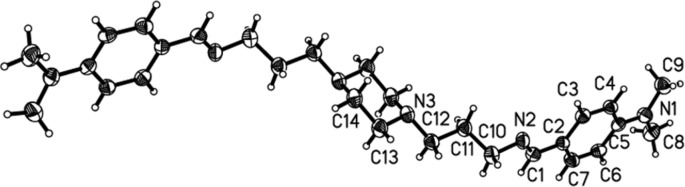
The molecular structure of (I). Displacement ellipsoids are drawn at the 30% probability level.

## supplementary crystallographic information

### Comment

Schiff bases and their metal complexes have been of great interest for many years due to their fascinating structural features, attactive properties and potential applications in many fields (Basak *et al.*, 2008; Jiang *et al.*, 2008; Xu *et al.*, 2008). While N,*N*'- disubstituted piperazines derivatives are antifilarial, antiamoebic and spermicidal agents (Yogavel *et al.*, 2003), therefore, studies on Schiff bases and their complexes derived from N,*N*'- disubstituted piperazines are of importance. As part of our work, the title compound,(I), a new tetradentate Schiff base ligand, are synthesized in our group and its crystal structure is reported here.

The molecular structure of (I) with atom-numbering scheme is shown in Fig.1. The bond length of C1—N2 (1.278 (7) Å) is equal to that of C1A—N2A, which is much shorter than the C—N single bond length (1.47 - 1.50 Å) and comparable with the reported values (Yogavel *et al.*, 2003; Thirumurugan *et al.*, 1998), indicating that the C—N bonds are double bonds. Two phenyl rings (C2—C7 and C2A—C7A) in (I) are perfectly parellel to each other. As for the piperazine moiety, the four atoms C13—C14—C13A—C14A are coplanar, and N3 atom or N3A atom lies above or below the mean plan by 0.6510 or -0.6510 Å. Furthermore, the plan makes dihedral angles of 129 ° with ring C13—N3—C14A or ring C13A—N3A—C14, indicating that the two rings are parallel and that the piperazine ring has a chair conformation just like other Schiff bases containing piperazine ring (Paital *et al.*, 2009; Thirumurugan *et al.*, 1998).

### Experimental

A solution of *N*,*N*'-bis(*N*-aminopropyl)-piperazine (1.5 mmol in 10 ml anhydrous methanol) was added dropwise with constant stirring to the solution of paradimethylaminobenzaldehyde (3 mmol in 15 ml anhydrous methanol) at 327 K for 3 h. The resulting mixture was filtrated. After cooling, the filtrate was evaporated at ambient environment. Several days later, the yellow crystals suitable for X-ray analysis were collected and washed with small amount of methanol and dried at room temperature (yield 77%).

### Refinement

H atoms were placed in calculated positions with C—H = 0.93–0.97 Å, and refined in riding mode with *U*_iso_(H)= 1.5 *U*_eq_(C) for methyl H atoms and *U*_iso_(H) = 1.2*U*_eq_(C) for the others.

### Crystal data

**Table:**

C_28_H_42_N_6_	*F*(000) = 504
*M**_r_* = 462.68	*D*_x_ = 1.118 Mg m^−^^3^
Monoclinic, *P*2_1_/*c*	Mo *K*α radiation, λ = 0.71073 Å
Hall symbol: -P 2ybc	Cell parameters from 683 reflections
*a* = 17.599 (2) Å	θ = 2.4–49.5°
*b* = 6.4146 (12) Å	µ = 0.07 mm^−^^1^
*c* = 12.6643 (18) Å	*T* = 298 K
β = 105.921 (3)°	Platelet, yellow
*V* = 1374.8 (4) Å^3^	0.15 × 0.09 × 0.07 mm
*Z* = 2	

### Data collection

**Table:**

Bruker SMART CCD area-detector diffractometer	961 reflections with *I* > 2σ(*I*)
Radiation source: fine-focus sealed tube	*R*_int_ = 0.088
graphite	θ_max_ = 25.0°, θ_min_ = 2.4°
φ and ω scans	*h* = −20→20
6788 measured reflections	*k* = −7→5
2416 independent reflections	*l* = −15→14

### Refinement

**Table:**

Refinement on *F*^2^	Secondary atom site location: difference Fourier map
Least-squares matrix: full	Hydrogen site location: inferred from neighbouring sites
*R*[*F*^2^ > 2σ(*F*^2^)] = 0.095	H-atom parameters constrained
*wR*(*F*^2^) = 0.298	*w* = 1/[σ^2^(*F*_o_^2^) + (0.0892*P*)^2^] where *P* = (*F*_o_^2^ + 2*F*_c_^2^)/3
*S* = 1.34	(Δ/σ)_max_ = 0.004
2416 reflections	Δρ_max_ = 0.24 e Å^−^^3^
155 parameters	Δρ_min_ = −0.17 e Å^−^^3^
0 restraints	Extinction correction: *SHELXTL* (Sheldrick, 2008), Fc^*^=kFc[1+0.001xFc^2^λ^3^/sin(2θ)]^-1/4^
Primary atom site location: structure-invariant direct methods	Extinction coefficient: 0.015 (5)

### Special details

**Table:**

Geometry. All e.s.d.'s (except the e.s.d. in the dihedral angle between two l.s. planes) are estimated using the full covariance matrix. The cell e.s.d.'s are taken into account individually in the estimation of e.s.d.'s in distances, angles and torsion angles; correlations between e.s.d.'s in cell parameters are only used when they are defined by crystal symmetry. An approximate (isotropic) treatment of cell e.s.d.'s is used for estimating e.s.d.'s involving l.s. planes.
Refinement. Refinement of *F*^2^ against ALL reflections. The weighted *R*-factor *wR* and goodness of fit *S* are based on *F*^2^, conventional *R*-factors *R* are based on *F*, with *F* set to zero for negative *F*^2^. The threshold expression of *F*^2^ > σ(*F*^2^) is used only for calculating *R*-factors(gt) *etc*. and is not relevant to the choice of reflections for refinement. *R*-factors based on *F*^2^ are statistically about twice as large as those based on *F*, and *R*- factors based on ALL data will be even larger.

### Fractional atomic coordinates and isotropic or equivalent isotropic displacement parameters (Å^2^)

**Table:**

	*x*	*y*	*z*	*U*_iso_*/*U*_eq_	
N1	0.9158 (3)	−0.7316 (8)	1.1684 (4)	0.0687 (16)	
N2	0.7447 (3)	0.0908 (8)	0.9057 (5)	0.0667 (15)	
N3	0.5629 (3)	0.4652 (7)	0.5991 (4)	0.0590 (14)	
C1	0.7396 (3)	−0.0090 (10)	0.9908 (6)	0.0625 (17)	
H1	0.7045	0.0408	1.0280	0.075*	
C2	0.7852 (3)	−0.1960 (9)	1.0339 (5)	0.0541 (16)	
C3	0.8387 (3)	−0.2867 (10)	0.9837 (5)	0.0598 (17)	
H3	0.8455	−0.2295	0.9194	0.072*	
C4	0.8822 (3)	−0.4629 (9)	1.0298 (5)	0.0563 (16)	
H4	0.9183	−0.5194	0.9961	0.068*	
C5	0.8728 (3)	−0.5567 (9)	1.1258 (5)	0.0539 (16)	
C6	0.8190 (3)	−0.4665 (9)	1.1741 (5)	0.0580 (16)	
H6	0.8113	−0.5255	1.2375	0.070*	
C7	0.7766 (3)	−0.2905 (10)	1.1299 (5)	0.0672 (18)	
H7	0.7414	−0.2333	1.1649	0.081*	
C8	0.9031 (4)	−0.8320 (10)	1.2662 (5)	0.083 (2)	
H8A	0.9375	−0.9505	1.2856	0.124*	
H8B	0.8492	−0.8767	1.2510	0.124*	
H8C	0.9145	−0.7346	1.3260	0.124*	
C9	0.9638 (4)	−0.8399 (10)	1.1115 (6)	0.083 (2)	
H9A	0.9889	−0.9563	1.1548	0.125*	
H9B	1.0033	−0.7471	1.0993	0.125*	
H9C	0.9312	−0.8890	1.0422	0.125*	
C10	0.6968 (4)	0.2755 (10)	0.8724 (5)	0.0704 (19)	
H10A	0.7309	0.3962	0.8784	0.084*	
H10B	0.6636	0.2962	0.9214	0.084*	
C11	0.6451 (4)	0.2571 (9)	0.7554 (5)	0.0665 (18)	
H11A	0.6095	0.1398	0.7500	0.080*	
H11B	0.6781	0.2311	0.7067	0.080*	
C12	0.5971 (3)	0.4555 (9)	0.7193 (5)	0.0637 (18)	
H12A	0.5548	0.4618	0.7547	0.076*	
H12B	0.6309	0.5758	0.7431	0.076*	
C13	0.5345 (4)	0.6762 (9)	0.5644 (5)	0.0710 (19)	
H13A	0.5778	0.7745	0.5873	0.085*	
H13B	0.4940	0.7152	0.5995	0.085*	
C14	0.5005 (4)	0.6860 (10)	0.4399 (5)	0.0678 (18)	
H14A	0.4803	0.8251	0.4189	0.081*	
H14B	0.5422	0.6586	0.4051	0.081*	

### Atomic displacement parameters (Å^2^)

**Table:**

	*U*^11^	*U*^22^	*U*^33^	*U*^12^	*U*^13^	*U*^23^
N1	0.081 (4)	0.071 (4)	0.055 (4)	0.017 (3)	0.020 (3)	0.013 (3)
N2	0.061 (3)	0.074 (4)	0.063 (4)	0.015 (3)	0.013 (3)	0.011 (3)
N3	0.054 (3)	0.060 (3)	0.063 (4)	0.007 (3)	0.015 (3)	0.012 (3)
C1	0.057 (4)	0.069 (4)	0.067 (5)	0.005 (3)	0.026 (3)	−0.007 (4)
C2	0.052 (3)	0.062 (4)	0.049 (4)	0.004 (3)	0.015 (3)	0.004 (3)
C3	0.062 (4)	0.067 (4)	0.052 (4)	−0.007 (3)	0.017 (3)	0.003 (3)
C4	0.057 (4)	0.062 (4)	0.052 (4)	0.003 (3)	0.019 (3)	0.001 (3)
C5	0.059 (4)	0.057 (4)	0.043 (4)	−0.007 (3)	0.009 (3)	0.003 (3)
C6	0.070 (4)	0.065 (4)	0.043 (4)	−0.005 (3)	0.023 (3)	0.000 (3)
C7	0.065 (4)	0.073 (5)	0.072 (5)	−0.002 (4)	0.033 (4)	0.003 (4)
C8	0.098 (5)	0.079 (5)	0.067 (5)	0.003 (4)	0.014 (4)	0.017 (4)
C9	0.097 (5)	0.069 (5)	0.086 (6)	0.013 (4)	0.027 (4)	0.004 (4)
C10	0.061 (4)	0.070 (5)	0.075 (5)	0.006 (3)	0.012 (4)	0.010 (4)
C11	0.069 (4)	0.067 (4)	0.068 (5)	0.012 (3)	0.026 (4)	0.015 (4)
C12	0.062 (4)	0.068 (4)	0.063 (5)	0.006 (3)	0.020 (3)	0.008 (3)
C13	0.075 (4)	0.063 (4)	0.072 (5)	0.013 (4)	0.017 (4)	0.013 (3)
C14	0.068 (4)	0.063 (4)	0.073 (5)	0.003 (4)	0.021 (4)	0.021 (4)

### Geometric parameters (Å, °)

**Table:**

N1—C5	1.378 (7)	C8—H8A	0.9600
N1—C9	1.432 (7)	C8—H8B	0.9600
N1—C8	1.466 (7)	C8—H8C	0.9600
N2—C1	1.278 (7)	C9—H9A	0.9600
N2—C10	1.448 (7)	C9—H9B	0.9600
N3—C13	1.468 (7)	C9—H9C	0.9600
N3—C14^i^	1.459 (7)	C10—C11	1.516 (8)
N3—C12	1.477 (7)	C10—H10A	0.9700
C1—C2	1.462 (8)	C10—H10B	0.9700
C1—H1	0.9300	C11—C12	1.527 (8)
C2—C7	1.404 (7)	C11—H11A	0.9700
C2—C3	1.400 (7)	C11—H11B	0.9700
C3—C4	1.399 (8)	C12—H12A	0.9700
C3—H3	0.9300	C12—H12B	0.9700
C4—C5	1.407 (7)	C13—C14	1.527 (8)
C4—H4	0.9300	C13—H13A	0.9700
C5—C6	1.387 (8)	C13—H13B	0.9700
C6—C7	1.384 (8)	C14—N3^i^	1.459 (7)
C6—H6	0.9300	C14—H14A	0.9700
C7—H7	0.9300	C14—H14B	0.9700
			
C5—N1—C9	122.3 (5)	N1—C9—H9B	109.5
C5—N1—C8	119.7 (5)	H9A—C9—H9B	109.5
C9—N1—C8	117.3 (5)	N1—C9—H9C	109.5
C1—N2—C10	119.0 (5)	H9A—C9—H9C	109.5
C13—N3—C14^i^	110.2 (5)	H9B—C9—H9C	109.5
C13—N3—C12	110.9 (5)	N2—C10—C11	111.5 (5)
C14^i^—N3—C12	112.2 (5)	N2—C10—H10A	109.3
N2—C1—C2	124.6 (6)	C11—C10—H10A	109.3
N2—C1—H1	117.7	N2—C10—H10B	109.3
C2—C1—H1	117.7	C11—C10—H10B	109.3
C7—C2—C3	117.4 (6)	H10A—C10—H10B	108.0
C7—C2—C1	120.0 (6)	C10—C11—C12	111.2 (5)
C3—C2—C1	122.7 (6)	C10—C11—H11A	109.4
C4—C3—C2	120.4 (6)	C12—C11—H11A	109.4
C4—C3—H3	119.8	C10—C11—H11B	109.4
C2—C3—H3	119.8	C12—C11—H11B	109.4
C3—C4—C5	121.7 (6)	H11A—C11—H11B	108.0
C3—C4—H4	119.2	N3—C12—C11	112.3 (5)
C5—C4—H4	119.2	N3—C12—H12A	109.1
N1—C5—C6	122.3 (6)	C11—C12—H12A	109.1
N1—C5—C4	120.3 (6)	N3—C12—H12B	109.1
C6—C5—C4	117.4 (6)	C11—C12—H12B	109.1
C7—C6—C5	121.3 (6)	H12A—C12—H12B	107.9
C7—C6—H6	119.3	N3—C13—C14	110.6 (5)
C5—C6—H6	119.3	N3—C13—H13A	109.5
C6—C7—C2	121.9 (6)	C14—C13—H13A	109.5
C6—C7—H7	119.1	N3—C13—H13B	109.5
C2—C7—H7	119.1	C14—C13—H13B	109.5
N1—C8—H8A	109.5	H13A—C13—H13B	108.1
N1—C8—H8B	109.5	N3^i^—C14—C13	111.6 (5)
H8A—C8—H8B	109.5	N3^i^—C14—H14A	109.3
N1—C8—H8C	109.5	C13—C14—H14A	109.3
H8A—C8—H8C	109.5	N3^i^—C14—H14B	109.3
H8B—C8—H8C	109.5	C13—C14—H14B	109.3
N1—C9—H9A	109.5	H14A—C14—H14B	108.0

Symmetry codes: (i) −*x*+1, −*y*+1, −*z*+1.
